# Balloon Pulmonary Angioplasty for Takayasu Arteritis and Peripheral Pulmonary Artery Stenosis Mimicking Chronic Thromboembolic Pulmonary Hypertension

**DOI:** 10.3390/life12111797

**Published:** 2022-11-06

**Authors:** Shigefumi Fukui, Yuko Shirota, Takao Nakano, Tsukasa Sato, Kaoru Hasegawa, Hisashi Kikuta, Takeyoshi Kameyama, Koji Kumagai, Tatsuya Komaru

**Affiliations:** 1 Department of Cardiovascular Medicine, Tohoku Medical and Pharmaceutical University, 15-1 Fukumuro 1-Chome, Miyagino-ku, Sendai 983-8536, Japan; 2 Department of Hematology and Rheumatology, Tohoku Medical and Pharmaceutical University, Sendai 983-8536, Japan

**Keywords:** chronic thromboembolic pulmonary hypertension, balloon pulmonary angioplasty, Takayasu arteritis, peripheral pulmonary artery stenosis

## Abstract

Balloon pulmonary angioplasty (BPA) has been reported to be effective and safe to an acceptable level in patients with distal-type, inoperable chronic thromboembolic pulmonary hypertension (CTEPH), resulting in improved long-term survival. However, evidenced treatment options and strategy including medical therapy of antithrombotic therapy, glucocorticoids, immunosuppressants, and pulmonary hypertension (PH)-specific therapies are scarce in patients with significant PH and right heart failure associated with Takayasu arteritis and peripheral pulmonary artery stenosis, both of which mimic CTEPH. Moreover, there has been still concern on safety and lack of established methodology in performing BPA for these conditions. In this report, we would like to review recent publications including several case reports and discuss the efficacy, safety, and suitable methods of BPA in this population.

## 1. Introduction

Chronic thromboembolic pulmonary hypertension (CTEPH) is characterized by stenosis and/or obstruction of pulmonary arteries due to organized thrombi, and in part, by a secondary small-vessel arteriopathy [1]. It is a life-threatening condition that leads to progressive right heart failure and a poor prognosis if left untreated [2]. Balloon pulmonary angioplasty (BPA) has been reported to be effective and safe to an acceptable level in patients with distal-type, inoperable CTEPH [3,4,5,6], who are not considered to be candidates for pulmonary endarterectomy (PEA). Furthermore, it is reported that BPA improves long-term survival, with a good safety profile, based on refined methodology [3,7]. Recently, the hybrid therapy of performing BPA before or after PEA, and also the combination of BPA with pulmonary hypertension (PH)-specific therapies, where two drugs are currently approved for CTEPH, have markedly progressed [8]. Thus, we can now expect good outcomes for patients with CTEPH owing to “thrombus” among group 4 PH in the latest ESC/ERS PH guidelines by appropriately applying PEA, BPA, and PH-specific therapies, compared with any other group of PH [9,10].

PH associated with pulmonary artery involvement in Takayasu arteritis (TA) and peripheral pulmonary artery stenosis (PPS) in adult patients, both of which mimic CTEPH in terms of pathophysiology and mismatch in lung ventilation-perfusion scintigraphy, are also categorized to group 4 PH in the latest PH guidelines [9]. TA is characterized by vasculitis invading from the adventitia, media, to intima, which leads to progressive arterial injury, with lesions such as intimal thickening, stenosis, occlusion, and aneurysm formation [11]. PPS is a rare and complex form, commonly associated with congenital rubella syndrome and genetic syndromes such as Williams–Beuren syndrome (WBS), and Alagille syndrome, although it is infrequently an isolated form [12]. PPS is characterized by multiple stenoses located sometimes diffusely in the pulmonary vascular tree [12]. Yanagisawa et al. reported that optical coherence tomography (OCT) imaging revealed abnormally thickened endothelium, unevenly thickened media, and coarctation of external elastic membrane (EEM) in patients with TA-PH and PPS, all of which are distinct findings from that in CTEPH [13]. By contrast, an intraluminal thrombus such as CTEPH was also detected by OCT, which may be the main target of BPA in TA-PH and PPS [13]. However, both TA-PH and PPS characterize “non-thrombotic” lesions such as EEM coarctation as a primary cause of stenosis and/or obstruction of pulmonary arteries etiologically. Therefore, efficacy of evidenced treatment options and strategy including medical therapy of antithrombotic therapy and PH-specific therapies, which have been customized for CTEPH, are not established in patients with significant PH and right heart failure associated with TA and PPS. Similarly, there has been still concern on a safety profile and lack of established methodology in performing BPA for these conditions.

In this report, we would like to review recent publications including several case reports and focus on the efficacy, safety, and suitable methods of BPA in adult patients with TA-PH and PPS.

## 2. Efficacy of BPA on TA and PPS

There have been several publications related to BPA for TA and PPS as follows in chronological order, shown in Table 1. In 1996, Kreutzer et al. reported 12 adult patients with isolated PPS from 17 to 51 years of age (mean, 36.2 ± 9.7 years) [14]. Eleven out of those patients were treated with BPA, in 9 of whom immediate procedural success was achieved [14]. After BPA, distal pulmonary arterial pressure increased ≥ 30% in 6 patients and RV pressure decreased > 30% in 5 patients [14]. At a mean follow-up period of 52 ± 32 months, 7 patients had sustained symptomatic improvement [14]. In 2003, Rothman et al. reported 4 adult women with isolated intralobar PPS from 18 to 63 years of age who underwent BPA [15]. After BPA, the mean ratio of right ventricular to aortic systolic pressure decreased from 0.92 to 0.62, with marked symptomatic improvement [15]. However, two of these patients had angiographic evidence of restenosis, which was subsequently treated with stent implantation [15]. The authors described that although BPA for intralobar PPS in adults was highly successful acutely, restenosis commonly took place within several months [15], which is inconsistent with the previous report by Kreutzer et al. In 2004, Tyagi et al. reported a patient with TA-PH due to severe proximal right pulmonary artery stenosis at the origin as well as total occlusion of the left subclavian, left common carotid, and right subclavian artery, who underwent percutaneous transluminal angioplasty (BPA) and stent implantation [16]. BPA improved the peak systolic gradient across the pulmonary artery stenosis from 70 to 12 mmHg and mean pulmonary arterial pressure (mPAP) from 52 to 30 mmHg, with increased arterial oxygen saturation [16]. In 2009, Qin et al. reported a total of 4 patients with isolated TA-PH who underwent percutaneous transluminal angioplasty (BPA), consisting of balloon angioplasty alone in one patient and balloon angioplasty and stent implantation in three patients [11]. After BPA, the pressure gradient improved from 58.3 ± 8.7 to 14 ± 3.2 mmHg and mPAP decreased from 48.5 ± 12.0 to 37.3 ± 6.0 mmHg [11]. During a follow-up period of 34.5 ± 15.8 months, the patient who was performed with balloon angioplasty alone developed a recurrence of symptoms 18 months after the procedure, whereas the other three patients continued to be asymptomatic [11]. In 2014, Dong et al. reported 14 consecutive patients with symptomatic pulmonary stenosis involved in TA, 22 lesions of whom were treated with percutaneous transluminal angioplasty (BPA), consisting of balloon angioplasty alone in 18 lesions and stent implantation in 4 [17]. Mean PAP significantly decreased from 53.4 ± 15.8 to 38.4 ± 12.7 mmHg immediately after intervention, with improved heart failure symptoms and 6-min walk distance (6MWD) at follow-up [17]. In 2015, Tonelli et al. retrospectively reported 6 adult patients with PPS and baseline mPAP of 32–60 mmHg at Cleveland Clinic, including Williams–Beuren syndrome in three patients [12]. The all patients were treated with balloon angioplasty alone, predominantly in the lobar arteries, with resultant improvements in mPAP that ranged from 16% to 46% in 5 patients, except one patient with no decrease in mPAP [12]. During a follow-up, no patient required reintervention [12]. In 2016, Yanagisawa et al. reported that there were 7 patients with PPS and 4 patients with TA-PH among 145 patients who underwent PTPA (BPA) in their institution [13]. After BPA (median, 5.0 sessions), mPAP and pulmonary vascular resistance (PVR) significantly improved from 46 to 34 mmHg and 10.5 to 4.6 Wood units, respectively [13]. Interestingly, in half of their cases, mPAP < 30 mmHg was not achieved, which is a distinct finding from their previous report demonstrating a mPAP of 21 mmHg after BPA in 83 patients with CTEPH [18]. These results suggest that the efficacy of BPA may be smaller for patients with TA-PH and PPS compared with that in CTEPH [13]. In 2020, Kamada et al. reported a patient of suspected TA with isolated pulmonary artery involvement complicated with severe PH, who underwent successful stent placement for stenosis in the right main pulmonary artery [19]. Mean PAP decreased from 45 to 22 mmHg and a concomitant increase in overall pulmonary blood flow, evaluated by 4D-flow cardiovascular magnetic resonance [19]. Ko et al. reported an adult patient with PPS complicated with Noonan syndrome, who was treated with total of four sessions of BPA [20]. After BPA, the mPAP improved from 27 to 17 mmHg, and the PVR dropped from 183 to 159 dyne s/cm^5^ with symptom alleviation [20]. In 2022, Huang et al. reported 32 consecutive patients (28 females, mean age, 42.8 ± 11.9 years) with TA-PH who underwent PTPA (BPA) [21]. After BPA, mPAP, PVR, and cardiac index all improved at follow-up from 49.7 ± 12.7 to 37.9 ± 9.6 mmHg, 10.1 ± 5.7 to 6.0 ± 2.3 Wood units, and 2.58 ± 0.72 to 2.96 ± 0.72 L/min/m^2^, respectively, along with ameliorated functional capacity such as World Health Organization functional class and 6MWD [21]. Moreover, pulmonary angiography revealed post-procedure restenosis in 64 (35.0%) lesions [21]. Most recently, Zhou et al. from China reported a total of 50 consecutive patients (41 females, mean age at diagnosis, 40 ± 14 years) with TA-PH who completed PTPA (BPA), which is a relatively large cohort compared to the previous studies [22]. Furthermore, they compared the PTPA group with the non-PTPA group, consisting of 21 patients who refused BPA and were treated with medical therapies alone, as control [22]. It is also worthy of note that they set up all-cause mortality, a hard event, for the primary outcome [22]. The 3-year survival rate of 93.7% in the PTPA group was significantly higher than 76.2% in the non-PTPA group [22]. The adjusted least-squares mean difference between the both groups was −12 mmHg (95% CI: −19 to −4; *p* = 0.003) in mPAP and −3.4 Wood units (95% CI: −5.1 to −1.7; *p* < 0.001) in PVR [22]. The restenosis rate (5 of 360 lesions; 1.4%) that occurred in 4 of 50 (8%) patients is extremely low [22]. It should be noted that the mPAP at reevaluation in the PTPA group was 38 ± 10 mmHg, which again did not achieve less than 30 mmHg [22].

## 3. Safety of BPA on TA and PPS

There is still concern on a safety profile in performing BPA for patients with TA-PH and PPS. Procedure-related complications include procedure-related death within 30 days, pulmonary artery injury such as pulmonary artery dissection and contrast extravasation caused by wire perforation and balloon overdilation, hemoptysis, hypoxemia, and reperfusion pulmonary edema (RPE). In 1996, Kreutzer et al. reported that one patient with severe PH at baseline, out of 11 patients treated with BPA, died shortly after BPA as a result of acute pulmonary edema and hemorrhage in the anterior segment of the right upper lobe associated with a distal pulmonary artery perforation [14]. They also reported that 6 patients had radiographic evidence of pulmonary edema in a dilated lung segment and that two patients had transient self-limited hemoptysis [14]. In 2003, Rothman et al. reported 4 adult women with isolated intralobar PPS, where all the patients developed transient RPE in lung segments distal to the intervention within 18 h after BPA, without any other significant complications related to BPA [15]. That edema resolved within a few days without specific therapy [15]. In 2009, Qin et al. reported 4 patients with isolated TA-PH who underwent BPA, where there was no complication associated with interventional therapy [11]. In 2014, Dong et al. reported 14 consecutive patients with TA-PH who underwent BPA [17]. Three patients (21.4%) had RPE, one of whom died due to respiratory failure 3 days after the procedure [17]. In 2015, Tonelli et al. reported 6 adult patients with PPS, all of whom were treated with BPA [12]. One patient developed a mild degree of RPE that resolved with diuretics in the course of 24 h [12]. In 2016, Yanagisawa et al. demonstrated that pulmonary artery injuries occurred just after balloon dilation in 14 BPA sessions in 7 patients with PPS and 4 patients with TA-PH, which were managed by prolonged dilation of the balloon at the proximal portion [13]. They described a significant difference in the prevalence of pulmonary artery injuries and hemoptysis after BPA between the patients in this cohort and previous CTEPH cohort (14 [25.5%] in 55 sessions vs. 35 [10.0%] in 350 sessions) [13]. None of the patients in this study showed clinically significant RPE [13]. In 2020, Kamada et al. reported a patient with suspected TA and severe PH who had only a small amount of bloody sputum without severe desaturation after BPA [19]. In 2022, Huang et al. reported 32 consecutive patients with TA-PH undergoing BPA [21]. Two (6.3%) patients developed RPE during the postoperative period, which was relieved after administration of furosemide, dexamethasone, and noninvasive ventilator adjuvant therapy [21]. Three (9.4%) patients experienced vascular dissection without extravascular leak, which was repeatedly compressed using a suitable balloon at 4–6 mmHg pressure for 3–5 min until disappearance of contrast agent retention [21]. Importantly, none of the patients died from BPA-related complications [21]. In the most recent publication by Zhou et al. from China, no periprocedural death occurred in 50 consecutive patients with TA-PH undergoing BPA [22]. Moreover, BPA procedure-related complications occurred in 28 of 150 BPA sessions (18.7%) [22]. Among them, pulmonary artery injury was the most common complication with an incidence of 18.0%, where mild to moderate hemoptysis was observed in 8 (5.3%) sessions [22]. Severe complications requiring noninvasive positive pressure ventilation occurred in only 1 of 150 total sessions (0.7%) [22].

## 4. Suitable Methodology of BPA on TA and PPS

In this chapter, we discuss suitable methodology of BPA on TA-PH and PPS by referring to the comments and issues published previously by those authors. We focus on the recent publications which were published after 2012, since when BPA has been performed based on refined methodology in patients with CTEPH, with a subsequent good safety profile [3]. In 2014, Dong et al. emphasized that it is important to select self-expandable stents due to the characteristics such as good flexibility and to perform BPA at the stable stage in terms of inflammation by vigilantly controlling inflammation during perioperative and long-term follow-up, to reduce risk of restenosis [17]. In 2016, Yanagisawa et al. recommended that selection of an optimally sized balloon catheter based on the precise identification of the EEM using intravascular imaging such as OCT may be crucial to avoid excessive dilation of the EEM in patients with PPS and TA-PH [13]. Thus, they recommend to perform BPA by targeting lesions accompanied by organized thrombi within a narrowed EEM [13]. In 2020, Kamada et al. advocated that performing BPA in a staged manner of balloon angioplasty followed by subsequent stent placement may result in gradually reduced mean PAP in a patient with suspected TA-PH, in order to avoid significant RPE [19]. Ko et al. reported that they used scoring balloons in a graded approach instead of stents or cutting balloons in an adult patient with PPS and Noonan syndrome, in order to avoid vessel injuries caused by excessive dilation of EEM, which may further lead to maintained dilatation of the lesion without restenosis at follow-up [20]. In 2021, Kim et al. reported in their review paper the treatment for peripheral pulmonary artery stenosis; PAS (PPS) that simple balloon angioplasty is the most basic therapeutic option for proximally located PPS [23]. Cutting balloon angioplasty is utilized for more dilation-resistant PPS vessels and for more distally located PPS [23]. They rated stent placement as the most effective option for the treatment of PPS because of a low rate of restenosis, and also recommended to reserve it for PPS vessels that are resistant to angioplasty [23]. In 2022, Huang et al. included 32 consecutive patients with TA-PH who had clinical remission with normal erythrocyte sedimentation rate and C-reactive protein level at baseline [21]. All the patients were administered dual antiplatelet therapy of aspirin and clopidogrel before BPA, and most patients were treated with PH-specific therapies such as bosentan [21]. They recommend from the technical viewpoint that balloon dilation should be stopped to avoid RPE when the distal mean PAP, measured by the pressure wire after each dilation, reaches 35 mmHg [21], which is based on the previous publication by Inami et al. [18]. Zhou et al. included 50 consecutive patients with TA-PH, where all baseline parameters in the PTPA group and the non-PTPA group were collected in the stable phase of TA, to reduce the influence of inflammation status with anti-inflammatory therapies of glucocorticoids and immunosuppressants [22]. Even after the procedure, small sustained doses of glucocorticoids were continued, regardless of erythrocyte sedimentation rate and C-reactive protein levels [22]. They argued that stent implantation should be performed only for proximal lesions to minimize the occurrence rate of in-stent restenosis [22]. For those patients who underwent stent implantation, dual antiplatelet therapy of aspirin and clopidogrel was prescribed for at least 6 months [22].

## 5. Discussion

To the best of our knowledge, this is the first review to systematically report the efficacy, safety, and suitable methods of BPA in adult patients with TA-PH and PPS in the current era when BPA has sufficiently evolved to an acceptable level in patients with inoperable CTEPH with refined methodology.

Regarding the efficacy of BPA in patients with TA-PH and PPS, most importantly, Zhou et al. demonstrated that the 3-year survival rate in the BPA group was significantly higher than that in the non-BPA group in the recent, relatively large cohort [22]. It may be in common that pulmonary hemodynamics such as mPAP, PVR, and cardiac index significantly improved after BPA, along with functional capacity such as World Health Organization functional class and 6MWD. However, the mPAP after BPA did not achieve less than 30 mmHg in most of the studies, except a few case reports, which suggests that the magnitude of BPA effects may be smaller for patients with TA-PH and PPS compared with that in CTEPH. This is probably attributed to the differences in the mechanisms of stenosis and/or obstruction of pulmonary arteries between TA-PH and PPS, and CTEPH. As Yanagisawa et al. demonstrated using OCT imaging, lesions in TA-PH and PPS consist of both EEM coarctation and an intraluminal thrombus [13]. Small-vessel arteriopathy may, in part, contribute to the loss of pulmonary vascular bed in TA-PH and PPS, like in case of CTEPH [1], although this has not yet been confirmed pathologically. In patients with CTEPH, intraluminal organized thrombi constitute the essential part in the pathogenesis, which requires lifelong, adequate anticoagulation therapy [1]. That is the reason why we perform BPA by targeting mainly such intraluminal thrombi and why BPA is highly effective to CTEPH. By contrast, in patients with TA-PH and PPS, EEM coarctation, a “non-thrombotic” lesion, constitutes the primary cause of stenosis and/or obstruction of pulmonary arteries etiologically. Therefore, if we perform BPA in patients with TA-PH and PPS with the same manner as CTEPH, we can easily intervene in intraluminal thrombi, but not sufficiently in EEM coarctation. This insufficient dilatation of EEM may be the reason why the efficacy of BPA was smaller for patients with TA-PH and PPS compared with that in CTEPH. Otherwise, if we try to tightly dilate EEM coarctation, it may lead to excessive dilation, resulting in increased risks such as pulmonary artery injuries and hemoptysis after BPA. To resolve these issues, several recent publications have adopted the use of intravascular imaging such as OCT and pressure wire, scoring balloons and cutting balloons, and a staged manner of BPA. In addition, these previous reports sometimes mentioned the strategy of antiplatelet therapy during a perioperative period, but scarcely anticoagulation therapy, which might be more important in the context of treating intraluminal thrombi. These points need to be further clarified in order to improve efficacy and safety concomitantly in patients with TA-PH and PPS, including the issues of elastic recoil and restenosis.

## 6. Conclusions

There have been increasing reports to demonstrate the efficacy of BPA in adult patients with TA-PH and PPS, including improved survival rate. However, the magnitude of hemodynamic improvement such as mPAP does not reach a comparable level to the case of CTEPH. Furthermore, the problems of recoil and restenosis after both balloon angioplasty and stent implantation remain to be solved. In recent publications, there are few cases to report severe complications requiring noninvasive positive pressure ventilation, invasive mechanical ventilation, or procedure-related death, suggesting an improved safety profile in patients with TA-PH and PPS. Finally, it may be important to use scoring balloons in order to avoid excessive dilation of EEM in PPS and to sufficiently stabilize inflammation with anti-inflammatory therapies before BPA in TA-PH. It may be also important in common for both conditions to adopt more careful management by using intravascular imaging such as OCT, pressure wire, and a staged manner of BPA. Randomized controlled studies will be needed to confirm these findings in the future.

## Figures and Tables

**Table 1:** The previous publications of BPA in TA-PH and PPS shown in chronological order.

Publication (Author)	Year	Patients (*n*)	Etiology	Treatment	mPAP (Average, mmHg)		Complication (Outcome)
					Before	After	
Kreutzer et al. [14]	1996	12 *	IPPS	BA	NA	NA	1 death 6 RPE
Rothman et al. [15]	2003	4	IPPS	2 BA 2 BA + stent	NA	NA	4 transient RPE
Tyagi et al. [16]	2004	1	TA	BA + stent	52	30	None
Qin et al. [11]	2009	4	ITA	1 BA 3 BA + stent	48.5 ± 12.0	37.3 ± 6.0	None
Dong et al. [17]	2014	14	TA	Majority BA 4 stents	53.4 ± 15.8	38.4 ± 12.7	1 death 3 RPE
Tonelli et al. [12]	2015	6	PPS	BA	46.8	35.6	1 RPE
Yanagisawa et al. [13]	2016	11	7 PPS, 4 TA	BA	46	34	14 PA injury No RPE
Kamada et al. [19]	2020	1	Suspected ITA	BA + stent	45	22	Small bloody sputum
Ko et al. [20]	2020	1	PPS	BA (scoring)	27	17	None
Huang et al. [21]	2022	32	TA	Majority BA 3 stents	49.7 ± 12.7	37.9 ± 9.6	6.3% RPE 9.4% PA injury
Zhou et al. [22]	2022	50	TA	Majority BA 14 stents	51 ± 13	38 ± 10	18.0% PA injury 5.3% hemoptysis 1 CPAP

* 11 out of 12 patients were treated with BPA. BPA, balloon pulmonary angioplasty; TA-PH, takayasu arteritis-associated pulmonary hypertension; PPS, peripheral pulmonary artery stenosis; mPAP, mean pulmonary arterial pressure; IPPS, isolated PPS; BA, balloon angioplasty; NA, not available; RPE, reperfusion pulmonary edema; ITA, isolated TA; PA, pulmonary artery; CPAP, noninvasive continuous positive airway pressure ventilation.

## Data Availability

Not applicable.
